# Phylogenetic and sequence profile analysis of Non-Ribosomal Polyketide Synthase-Adenylation (NRPS)domain from *Actinobacterium dagang 5*

**DOI:** 10.6026/97320630017809

**Published:** 2021-09-30

**Authors:** Deepika Thandayuthapani, Nivetha Chinnappa, Arjunan Annavi, Muthusevam Manickam

**Affiliations:** 1 Department of Biotechnology, Bharathidasan University, Tiruchirappalli-620020, Tamilnadu, India

**Keywords:** *Actinobacterium dagang 5*, NRPS, Adenylation domain, Metabolite profiling, Gas chromotography

## Abstract

This study aims to find out the mapping of bioactive compounds by combinational analysis of regulatory machinery pattern study and metabolomics approach. In which we isolated a highly potent *Actinobacterium dagang 5* from Gulf of Manner, which shows broad-spectrum activity against several pathogens. So the isolate was used for overall metabolic profiling studies on crude extract and phylogeny pattern analysis of NRPS A-domain, which is an important gene clusters and plays vital role in production of bioactive metabolites. The result suggests that *Actinobacterium dagang 5* has the potential to produce a new type of antibacterial compounds.

## Background:

The increase in urge of new antibiotics due to the rapid spread of antibiotic-resistant pathogenic organism, which causing life-threatening infections and the chemically synthesized drugs cause side effects [1]. These reasons increase to invade the natural product drug discovery [2]. The marine environment is largest resource with taxonomically diverse bacterial groups, which are unique in nature and they are extremities, with potential and novel secondary metabolites [3,4]. The marine environment microbes and producing compounds are not exhibited in the terrestrial sources [5]. So the research has been focused on the screening on marine sediment derived microorganisms [6], numerous of antibiotics are isolated from marine source, of which one- third of drug are isolated from Actinomycetes [7]. This has ability to produce the novel secondary metabolites such as antimicrobial, anticancer, anti-inflammatory agents with novel structure and properties. Therefore isolation of marine actinomycetes and cultivating in laboratory is tedious process, later optimizing its efficient source and conditions it will provide us ample scope the investigation of the biologically active metabolites. Streptomyces are most valuable soil inhabiting Gram-positive bacteria, filamentous in nature with vast source of novel biologically active compounds with unique structure and properties [8,9,10]. The new techniques are arrived for isolation, cultivation and screening to increase the diversity of cultivable isolates. Various pre-treatment was employed to enrich the isolation of actinomycetes from other genera; Air-dry treatment of soil is universal method to increase the number of mycelium forming actinomycetes. Sampling sites, sampling strategy, geographic and habitat plays essential role in the isolation of actinomycetes [11]. Genome Mining is integrated approach, of which bioinformatics, molecular biology, natural product chemistry combined together to access the metabolic product of gene cluster [12]. In general the early reports denote that the Streptomyces has High G+C content, which impact on the short reads with higher error [13]. The Streptomyces have the biosynthetic gene clusters, which is the machinery for production of secondary metabolites [14]. The Polyketide Synthase (PKS) and Non-Ribosomal Polyketide Synthase (NRPS) genes place vital role in prediction of chemical nature of compound. Thus the genome mining approach paves the new path in identification in degree of novelty of compound.

## Materials & Methods:

### Isolation and extraction of intracellular and extracellular metabolite:

The pure culture of the *Actinobacterium dagang 5* was transferred aseptically to the 250 ml of seed medium in 1000 ml Erlenmeyer flask and incubated at 30°C for 7days on the rotary shaker at 180 rpm.

### Isolation of extracellular metabolite:

The cell free supernatant was collected separately and filtered with cheesecloth. The obtained filtered culture was centrifuged at 10000rpm for 15min and the supernatant undergone to polar solvent extraction using ethyl acetate in the ratio of 1:1 (v/v) followed by vigorous shaking for 30min.The ethyl acetate fractions were pooled and evaporated under vacuum using a roto evaporator (BUCHI, Switzerland) [15,16]. Then the resultant crude extract was subjected to Gas chromatography.

### Isolation of intracellular metabolite:

The mycelia were obtained and liquid nitrogen was added at -25°C and incubated for 3 sec and lyophislised at -20°C. Then sample was undergone to methanol extraction with the equal volume of solvent with vigorous shaking for 30 min and vacuum dried. Then the resultant crude extract was subjected to Gas chromatography [17,18].

### Extraction of Genomic DNA:

The pure culture of the *Actinobacterium dagang 5* was transferred aseptically to the 250 ml of seed medium in 1000 ml Erlenmeyer flask and incubated at 30°C for 7days on the rotary shaker at 180 rpm. The Mycelia was harvested by centrifuging for 10 min at 10000 rpm in 4°C. The SET buffer (25 mM TrisHCl, 25 mM EDTA, pH 8, 0.3 M Sucrose) and 3 µL of lysozyme (20 mg/mL) was added and vortexed vigorously followed by incubation at 37°C for 45 mins. 600 µL of 10% SDS was added and incubated at 56°C for 15mins. Later 3 µL of RNase was added and incubated at 37°C for 15mins. 2mL of Phenol: chloroform: isopropanol (25:24:01) and centrifuged at 10000 rpm in 4°C for 10 mins, the aqueous layer was transferred carefully to the sterile tube and precipitate with ice cold absolute ethanol, then the mixture was kept in -80°C for 30 mins, the precipitate was transferred to the sterile tube and centrifuge for 10000 rpm for 10mins, The pellet obtained was washed with 70% ethanol and dried in room temperature. Finally the pellet was dissolved 200 µL of TE buffer (10 mM Tris, 1 M EDTA, pH 8). Transferred and the DNA was checked in 1% Agarose (Agarose gel electrophoresis).

### Bacterial strains, plasmid and cultural conditions:

*E.coli* DH5α strain was used for DNA cloning and plasmid propogation. *E.coli* were cultured in the Luria broth at 37°C with appropriate antibiotics. The cloning vector pGEM®-T was purchased from Promega, India.

### NRPSs amplification, cloning and sequencing:

The NRPS gene was amplified from genomic DNA from potential strain *Actinobacterium dagang 5* using degenerate primer ADEdom 5'- CCA ACS GGC NNN CCS AAG GGC GT 3' and ADEdom 5'- ACC CTC SGT SGG SCC GTA -3', which is the 450bp fragment representing adenylation domain region of NRPS gene. The codes "N","S","B" and "Y" represent any base (A/T/G/C), G /C , (T/G/C) and C/T respectively.

PCR was performed with the 50 µl reaction mixture of containing 1 µM of each primer, 1µl extracted DNA, 23 µl sterile distilled H2O and 10% (v/v) of DMSO to the final volume of PCR premix (Emerald, Takara, Japan). Amplification were performed with the thermo cycler, the polymerase chain reaction as follows 5 min at 95°C and followed by 40 cycles of 1 min at 94°C, 1 min at 60°C and 1.5min at 72°C followed by 10 min final extension at 72°C.The amplified products were analyzed by electrophoresis in 1% (w/v) agarose gels and purified by DNA extraction kit (Quiagen, India). The purified PCR product was ligated with pGEM® -T vector according to the manufactures instructions. Ligation mixture was transformed to *E.coli* DH5α strain using electrophoration technique, the plasmid DNA from the transformants was isolated using HiYield TM plasmid mini kit (RBC, Korea). To confirm the clone, the PCR was proceed with Primer pair of T7 promoter 5’ TAA TAC GAC TCA CTA TAG GG 3' and SP6 promoter 5' GAT TTA GGT GAC ACT ATA G 3'. PCR was performed with the 50 µl reaction mixture of containing 1 µM of each primer, 2 µl extracted DNA, 23 µl sterile distilled H2O and 10% (v/v) of DMSO to the final volume of PCR premix (Emerald , Takara, Japan). Amplification were performed with the thermo cycler, the polymerase chain reaction as follows 5 min at 95°C and followed by 35 cycles of 1 min at 94°C, 1 min at 54°C and 1.5min at 72°C followed by 10 min final extension at 72°C.The amplified products were analysed by electrophoresis in 1% (w/v) agarose gels and purified by DNA extraction kit (Quiagen, India). Sequencing was performed on ABI 310 automatic DNA sequencer using the SP6 and T7 promoter primer.

### Phylogenetic analysis of NRPS adenylate domain:

The sequences were aligned using BLAST tool, multiple alignment was performed using the CLUSTAL_X and the phylogenetic tree was constructed using MEGA 6.0 software.

## Results and Discussion:

The intra and extracellular crude extract was evaluated with the GC-MS, where obtained 17 and 12 peaks in chromatogram for the intracellular and extracellular crude extracts receptively. The peak was evaluated with the NIST database. The intracellular crude extracts subjected to GC-MS analysis revealed the presence of 17 peaks noted at the retention time of 5.932 (C1), 7.354(C2), 11.161 (C3), 11.265 (C4), 11.542 (C5), 13.110 (C6), 13.258 (C7), 13.968 (C8), 16.106 (C9), 16.498 (C10), 16.627 (C11),17.987 (C12), 18.042 (C13), 18.400 (C14), 21.202 (C15),21.435 (C16), 21.767 (C17) (Figure 1 - see PDF). Further examination of these peaks by MS showed molecular ions at m/z 69(C1); 44 (C2); 42 (C3); 36(C4); 61 (C5); 79 (C6); 79 (C7); 68 (C8); 80 (C9); 75 (C10); 47 (C11); 38 (C12); 73 (C13); 86 (C14); 93 (C15); 110 (C16); 82 (C17)of molecular weights. According to the available library data, NIST-MS search (included with NIST'02 mass-spectral library, Agilent p/n G1033A) compounds C1, C2, C3, C4, C5, C6, C7, C8, C9, C10, C11,C12, C13, C14, C15, C16 and C17 were identified asl-Alanine, N-methoxycarbonyl-, methyl ester, Dianhydromannitol, l-Alanine, N-methoxycarbonyl-, methyl ester, l-Alanine, Nmethoxycarbonyl-, methyl ester, Phenol, 3,5-bis(1,1-dimethylethyl), l-Alanine, Nmethoxycarbonyl-, methyl ester, l-Alanine, N-methoxycarbonyl-, methyl ester, Tetradecanoic acid, methyl ester, Hexadecanoic acid, methyl ester, n-Hexadecanoic acid, Dibutyl phthalate, Sarcosylsarcosine, N-methoxycarbonyl-, propyl ester, Methyl stearate, Hexanoic acid, heptadecyl ester, Hexadecanoic acid, 2-hydroxy-1- (hydroxymethyl)ethyl ester Benezenedicarboxylic acid respectively.

The extracellular crude extracts subjected to GC-MS analysis revealed the presence of 12 peaks noted at the retention time of 12.584 (C1), 13.589(C2), 14.142 (C3), 14.400 (C4), 14.685 (C5), 15.522 (C6), 15.641 (C7),16.245 (C8), 16.566 (C9), 16.498 (C10), 21.448 (C11),21.686 (C12) (Figure 2 - see PDF). Further examination of these peaks by MS showed molecular ions at m/z 81(C1); 70 (C2); 52(C3); 80 (C4); 91 (C5); 64 (C6); 74 (C7); 72(C8); 98 (C9); 62 (C10); 68 (C11); 67 (C12) of molecular weights. According to the available library data, NIST-MS search (included with NIST'02 mass-spectral library, Agilent p/n G1033 A) compounds C1, C2, C3, C4, C5, C6, C7, C8, C9, C10, C11andC12were identified as Diethyl Phthalate, 1-Tetradecanol, Acrylate, 3-Methyl-1,4-diazabicyclo [4.3.0]nonan2,5-dione,N-acetyl- Hexanoic acid, dodecyl ester, 1,4-Diaza-2,5-Dioxobicyclo [4.3.0]nonane, 3-Iso Buty Hexahydropyrrolo[1,2-A] Pyrazine-1,4-Dione, Caffeine, Pyrrolo[1,2-a]pyrazine-1,4-dione, hexahydro-3-(2-methylpropyl), 1,2-Benzenedicarboxylic acid, butyl octyl ester, 2,2,4,4,6,6,8,8,10,10,12,12,14, 14,16,16,18,18,20,20-Icosamethylcyclodecasiloxane, Cyclododecasiloxane, TetracosaMethyl, Bis (2-ethylhexyl) phthalate. NCBI PubChem bioassay database (https://pubchem.ncbi.nlm.nih.gov) exhibits that among the overall 29peaks, nearly most of the compounds indicating the presence of bioactive constituents have been previously reported for their antimicrobial activity.

The 450bp encoding the adenylation domain region of NRPSs region was successfully amplified from the genomic DNA of *Actinobacterium dagang 5*. The amplified product was cloned in pGEM® -T vector, to identify the positive clones they are subjected to the PCR screening. The amplified product from pGEM® -T vector as template of size 560bp. Later the positive clone was sequenced with SP6 and T7 promoter. The amino acid sequence corresponding to obtained Adenylation domain region of NRPSs gene of Actinobacterium.dagang5 shows conserved motif region (Figure 3 - see PDF). The three-motif region was identified in the 450bp of amplified A-domain region of NRPS gene. The three major motif regions are of TGxPKGV, FD and NxYGPTE, which has been reported earlier in Actinomycetes. The resulting shows motif shows high similarities those available in gene bank, finally the results indicates that the isolates have natural compounds.

In earlier reports NRPS Adenylation domain region was isolated from Antarctic sediments [19] and temperate forest Gerenzano, Italy [20]. The presence of NRPS A domain region gives the valuable genome based information regarding the anti microbial activity of isolates [21], it gives valuable information regarding the bioactive compounds present in the isolates by comparing the high similarities organism. So it paves way to predict the peptide antibiotics of the novel isolate. It has been reported for the past few decades, regarding the studies on the biosynthetic gene cluster or secondary metabolite production machinery to know about the functional roles of gene in production of bioactive compound [22]. So in our present study, we have attempted the genome based approach before going for the fermentation process. Phylogenetic analysis of the NRPS A domain region pinpoint the bioactive compound from phylogentically distant strain, so we can narrow down our research on particular assay such as antibacterial assay or antifungal etc., so its time consuming process.

NRPSs A domain based phylogenetic tree (Figure 4 - see PDF) shows that the NRPS A domain region shares higher similarity with those available in data bank. Sequence shows 85% similarity with Streptomyces sp. A188 and Nocardiopsis sp and the result suggest the isolate has the potential bioactive compounds with antibacterial properties to relate with the functional genes. By combining the both metabolite profiling data and evolutionary analysis of the NRPS Adenylation domain the study reflects that the isolate *Actinobacterium dagang 5* is strong bioactive metabolite producer.

## Conclusion:

As per literature studies the marine actinomycetes are one of the most of bioactive compound producers, the isolate Actinobacteriumdagang5 exhibit strong antibacterial activity, so the isolate is intended to metabolite profiling and phylogentic analysis of NPRS adenylation domain region. By merging the obtained data of both studies reveals that the isolate would exhibit the broad-spectrum antimicrobial metabolite.
